# Toward an accurate prediction of inter-residue distances in proteins using 2D recursive neural networks

**DOI:** 10.1186/1471-2105-15-6

**Published:** 2014-01-10

**Authors:** Predrag Kukic, Claudio Mirabello, Giuseppe Tradigo, Ian Walsh, Pierangelo Veltri, Gianluca Pollastri

**Affiliations:** 1 School of Computer Science and Informatics, Complex and Adaptive Systems Laboratory, University College Dublin, Belfield, Dublin 4, Ireland; 2 Department of Clinical and Experimental Medicine, University Magna Græcia of Catanzaro, Catanzaro 88100, Italy; 3 Department of Biology, University of Padua, Viale G. Colombo 3, I-35131 Padova, Italy; 4 Chemistry Department, University of Cambridge, Lensfield Road, Cambridge CB2 1EW, UK

**Keywords:** Distance map, Recursive neural network, *ab initio* predictor, Template-based predictor

## Abstract

**Background:**

Protein inter-residue contact maps provide a translation and rotation invariant topological representation of a protein. They can be used as an intermediary step in protein structure predictions. However, the prediction of contact maps represents an unbalanced problem as far fewer examples of contacts than non-contacts exist in a protein structure.

In this study we explore the possibility of completely eliminating the unbalanced nature of the contact map prediction problem by predicting real-value distances between residues. Predicting full inter-residue distance maps and applying them in protein structure predictions has been relatively unexplored in the past.

**Results:**

We initially demonstrate that the use of native-like distance maps is able to reproduce 3D structures almost identical to the targets, giving an average RMSD of 0.5Å. In addition, the corrupted physical maps with an introduced random error of ±6Å are able to reconstruct the targets within an average RMSD of 2Å.

After demonstrating the reconstruction potential of distance maps, we develop two classes of predictors using two-dimensional recursive neural networks: an *ab initio* predictor that relies only on the protein sequence and evolutionary information, and a template-based predictor in which additional structural homology information is provided. We find that the *ab initio* predictor is able to reproduce distances with an RMSD of 6Å, regardless of the evolutionary content provided. Furthermore, we show that the template-based predictor exploits both sequence and structure information even in cases of dubious homology and outperforms the best template hit with a clear margin of up to 3.7Å.

Lastly, we demonstrate the ability of the two predictors to reconstruct the CASP9 targets shorter than 200 residues producing the results similar to the state of the machine learning art approach implemented in the Distill server.

**Conclusions:**

The methodology presented here, if complemented by more complex reconstruction protocols, can represent a possible path to improve machine learning algorithms for 3D protein structure prediction. Moreover, it can be used as an intermediary step in protein structure predictions either on its own or complemented by NMR restraints.

## Background

The ability to correlate the function of a protein and its three-dimensional (3D) structure is a challenge of fundamental importance in computational biology. However, deciphering this structure-function relationship requires the availability of much more structural data than experimental methods can currently provide. The lack of structural data, in contrast to the wealth of existing proteins sequences [1-3], has been addressed in the last three decades by constructing different computational models for predicting protein 3D structures from primary amino acid sequence information.

Existing structure prediction models are typically divided into two broad categories: template-based and *ab initio*. Template-based models utilize sequence and structure similarity between an unknown protein, the so-called ‘target’ , and known structures, termed ‘templates’ , fathomed to be homologous to the target. This category of models has become increasingly accurate in predicting the structures of globular proteins over the last years [4-6]. However, the accuracy of template-based models strongly relies on the degree of similarity between the target and its templates, thus preventing its application to a significant fraction of unannotated proteins. On the contrary, the *ab initio* models are usually employed for proteins that have no detectable homology to proteins of known structure and therefore these models are not nearly as accurate as their template-based counterparts [4-6]. However, the most prevalent *ab initio* models still utilize known protein structures to some degree, i.e. small structural fragments with the strong sequence-structure relationship [7]. As such, structural fragments are used as building blocks in reconstructing the complete structure of the target protein. This process is typically governed by the use of statistical constraints [7], force fields [8] and/or NMR spectra [9]. Only recently, models that use only evolutionary constraints have emerged [10,11].

In the absence of a reliable solution to the protein structure prediction problem, some research groups have focused on solving simplified problems such as the prediction of protein structural features [12-14]. The most frequently predicted structural features are: protein secondary structure, relative solvent accessibility, contact density and contact maps. Once these protein features have been obtained, they can be used to guide the reconstruction process implemented by a simple geometric-based algorithm [15]. Among protein structural features, contact maps have been proposed as an intermediate step in assembling the unknown protein 3D structure from its amino acid sequence [16-22]. Contact maps are usually predicted in binary form, i.e. they contain information about the mutual contact/non-contact between a pair of residues or atoms, where a contact is defined according to some distance cut-off criterion. Even though contact maps do not contain all information about a protein, such as chirality, they do provide a good overall topology of the protein structure. In fact, it has been argued that a contact map with an adequate threshold for a contact provides sufficient information to reconstruct native or near-native structure [15]. Beyond the prediction of protein structures, protein contact maps have been also used in a number of other structural tasks, for instance as protein fingerprints for rapid prediction of protein structures similarity [23-25], in the prediction of protein folding rates [26], protein disorder [27] and inter-domain contact regions [28].

The prediction of contact maps represents an unbalanced problem as far fewer examples of contacts than non-contacts exist in a protein structure. Therefore, it is not surprising that the prediction accuracy of contact maps is still low despite years of attempts [29]. To mitigate the unbalanced nature of contact/non-contact predictions, a method that predicts 4-class distance maps has recently been introduced [30]. The 4-class distance map has been shown to improve both the residue contact prediction and the C_α_-trace reconstruction compared to its binary counterpart [30].

In this study we explore the possibility of completely eliminating the unbalanced nature of the contact map prediction problem by predicting real-value distances rather than contacts. In particular, we predict an inter-residue distance map, i.e. a 2D symmetric matrix whose entry (*i, j*) represents the distance between residues *i* and *j* along the protein sequence. Unlike binary and multi-class contact maps where classifying distances into a few states is somewhat arbitrary, a distance map contains real distances between protein residues. As a consequence, not only is the unbalanced nature of the contact prediction eliminated, but also the poor approximation of those distances in the vicinity of the class boundaries.

The possibility of predicting inter-residue distances has been relatively unexplored in the past. To our knowledge, only a few studies concerning inter-residue distances exist in the literature where a limited number of distance restraints have been predicted [31,32] or only an inter-residue distance distribution has been studied [33,34]. Here, for the first time, we explore the possibility of predicting full inter-residue distance maps. In the first part of this study, we examine the ability of native distance maps to reconstruct near native protein structures. We show that native distance maps give rise to more accurate C_α_-traces than native multi-class and binary contact maps even when a random error of 6Å is added to the maps. Then, we describe two classes of predictors developed here that are based on two-dimensional recursive neural networks (2D RNN): an *ab initio* predictor, which relies only on the protein sequence and evolutionary information, and a template-based predictor in which additional structural homology information is provided. In addition, we report on the average RMSD between the native and predicted distance maps obtained as outputs of the *ab initio* and template-based predictors. In the final part of this study, we test the two predictors in reconstructing protein structures of the CASP9 targets and briefly gauge the quality of the reconstructed traces.

## Results

### Reconstruction of Cα-traces using native contact and distance maps

Our first question in this study is concerned with the ability of distance maps to adequately reconstruct C_α_-traces. With this in mind, we use native maps extracted from 93 solved 3D structures of the CASP7 targets. The CASP7 targets represent an ideal dataset for this purpose due to their intermediate length (the average number of residues per structure of 192 with 85% of structures consisting of 100 to 300 residues) and the variety of protein structural motifs they include. As a reconstruction protocol we use a simple procedure described in detail within the Methods section. Using this reconstruction procedure we only enforce the constraints encoded in the map and very basic geometrical rules, such as the distances between neighbouring C_α_ atoms, the geometry of α-helices and β-strand lengths. As a corollary, any improvement in reconstruction results using distance over coarse maps derive from the wealth of structural constraints encoded in the distance maps compared to their coarse counterparts.

In the following, we compare the quality of reconstructions based on binary, 4-class and distance maps when experimental constraints are known, that is the maps are native. Binary contact maps are provided with a distance cut-off of 12Å between a contact and a non-contact. Even though a threshold of 8Å is commonly used in the CASP experiments [35], the results of a 12Å threshold are presented here, as this threshold leads to more accurate reconstructions in our tests, in agreement with the conclusions from in [30,36]. The 4-class maps are identical to those in [30] and include three threshold values: 8Å, 13Å and 19Å.

For each protein in the CASP7 dataset we run 10 folding simulations and select the best reconstructed structure. As a measure of quality we use root mean square deviation (RMSD), global distance test total score (GDT_TS) and template modelling score (TM-score) between the predicted and native structure. Unlike the RMSD measure which is based on a single general superposition between two structures, the GDT algorithm is based on multiple local superpositions [37]. In particular, the GDT_TS score reports the largest, not necessarily continuous, set of ‘equivalent’ residues that deviate by no more than a particular distance cut-off (1Å, 2Å, 4Å and 8Å). TM-score [38], on the other hand, is a measure sensitive to the correctness of the global topology rather than to the local structural errors. It lies in the [0,1] interval, with values above 0.4 indicating a model with a roughly correct topology, and values below 0.17 indicating random prediction regardless of the protein size [38]. The RMSD, GDT_TS and TM-score for the best simulation are averaged over all 93 CASP7 proteins and are reported in Table 1.

**Table 1:** **Reconstruction of C**_**α**_**-traces from native and non-native maps**

**Maps**	**RMSD [Å]**	**GDT_TS**	**TM-score**
Binary	4.38 (0.90, 14.98)	0.72 (0.42, 0.96)	0.77 (0.29, 0.97)
Binary ± 3Å	4.05 (1.50, 12.44)	0.64 (0.36, 0.82)	0.74 (0.42, 0.90)
Binary ± 6Å	4.26 (2.54, 9.78)	0.53 (0.32, 0.67)	0.64 (0.29, 0.78)
4-Class	1.04 (0.47, 6.90)	0.94 (0.73, 1.00)	0.95 (0.79, 0.98)
4-Class ± 3Å	1.41 (0.88, 6.80)	0.85 (0.67, 0.93)	0.90 (0.72, 0.96)
4-Class ± 6Å	2.25 (1.53, 4.08)	0.70 (0.56, 0.81)	0.81 (0.57, 0.88)
Distance	0.48 (0.22, 0.87)	0.99 (0.94, 1.00)	0.99 (0.94, 0.998)
Distance ± 3Å	0.96 (0.66, 1.46)	0.92 (0.85, 0.98)	0.94 (0.73, 0.99)
Distance ± 6Å	1.62 (1.03, 4.20)	0.81 (0.57, 0.88)	0.87 (0.48, 0.96)

The reconstruction of C_α_-traces derived from binary contact maps, 4-class contact maps and distance maps. The native maps and the maps with a random error of 3Å and 6Å are used with the basic reconstruction protocol. Average RMSD [Å], GDT_TS [fraction] and TM-score, along with their range (min, max) are reported using the CASP7 targets.

If more distance constraints are provided to the simple reconstruction algorithm, it is expected that more accurate structural predictions would follow. Therefore, it is not surprising that the reconstruction based on the native binary maps produces structures of the lowest quality, with an average RMSD of 4.38Å, a GDT_TS of 72% and a TM-score of 0.77 (Table 1). Native 4-class maps include more distance constraints than their binary counterparts and lead to structures with an average RMSD of 1.04Å, a GDT_TS of 94% and a TM-score of 0.95. Finally, the reconstruction based on the native distance maps that encode the real-value inter-residue distances is able to reproduce even more accurate structures having an average RMSD of only 0.48Å, a GDT_TS of 99% and a TM-score of 0.99. The main problem experienced by the binary and 4-class contact maps in reconstructing the near-native structures is observed in the proteins with structurally disordered segments, e.g. the long coils in a 250-residue structure of the T0381 target, PDB ID: 2I2A (RMSD_4-class_ = 6.9Å, RMSD_binary_ = 14.6Å) and a 100-residue structure of the T0309 target, PDB ID: 2H4O (RMSD_4-class_ = 2.5Å, RMSD_binary_ = 12.0Å). On the other hand, the reconstruction protocol with distance maps is able to reproduce the two structures with an RMSD of 0.5Å and 0.4Å, respectively. Furthermore, the reconstruction protocol and the distance maps of all other structures give consistent results with an RMSD being in the narrow range between 0.22Å and 0.87Å.

A non-native distance map conveys more structural information than its coarse counterparts and, thus, is expected to convey more errors at the same time. Therefore, in the following we set out to investigate the impact of distance constraints with various degrees of errors on the used geometric reconstruction protocol. To this end we generate binary, 4-class and distance native maps with a random error of ±3Å and ±6Å for the same CASP7 targets, and further use them in the reconstruction protocol (Table 1). When an error of ±3Å is added to the maps, the accuracy of the reproduced structures decreases slightly using all of the three map types. Distance maps still produce the best reconstruction results (RMSD = 0.96Å, GDT_TS = 92%, TM-score = 0.94), followed by 4-class maps (RMSD =1.41Å, GDT_TS = 85%, TM-score = 0.9) and binary maps (RMSD = 4.05Å, GDT_TS = 64%, TM-score = 0.74). Here, it is interesting to point out that the lowest RMSD in the dataset always deteriorates when the error is included in the maps, whereas the largest RMSD value in the dataset and the mean RMSD can even improve. On the other hand, the corresponding values of GDT_TS and TM-score (max, min and mean values) almost always deteriorate with an increase of the error. This confirms that for low accuracy models RMSD is no longer a meaningful measure of the quality of the models and GDT_TS and TM-score should therefore be given precedence.

Finally, we increase a random error to ±6Å and calculate the accuracy of the reconstructions. As expected, the accuracy of the reconstructed structures decreases further, but the folds in most structures remain essentially the same. Even with an error as large as ±6Å, distance maps still yield more accurate structures than 4-class and binary maps. Specifically, distance maps produce structures with an average RMSD of 1.62Å, a GDT_TS of 81% and a TM-score of 0.87; 4-class maps produce structures with an average RMSD of 2.25Å, a GDT_TS of 70% and a TM-score of 0.81; binary maps produce structures with an average RMSD of 4.26Å, a GDT_TS of 53% and a TM-score of 0.64. Even though distance maps and 4-class maps with a ±6Å error are still able to reproduce accurate folds for the proteins, the possible application of these models in structural studies is more limited beyond this level of error.

### Distance map prediction

**Figure 1:**
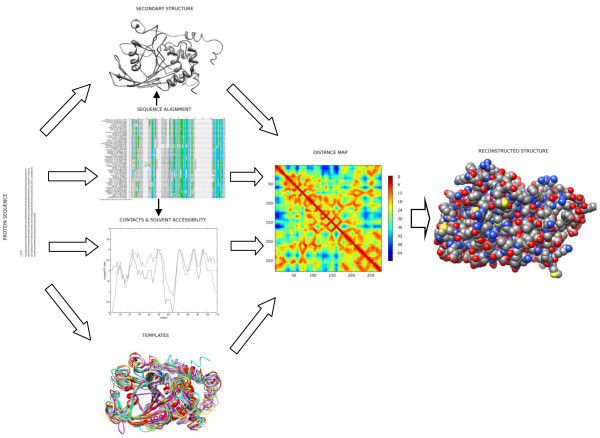
**The workflow of the algorithm.** The overall workflow of the protein structure prediction algorithm on the example of Protein-Tyrosine Phosphotase 1B (PDB ID: 2HNP). The first stage includes predictions of protein secondary structure, contact density and relative solvent accessibility, as well as finding and ranking appropriate templates. Using the structural features from the first step, the distance map is predicted in the second stage. In the last stage the actual 3D coordinates of all atoms and residues in the structure are reconstructed.

After establishing the potential of distance maps in protein 3D structure predictions, we set out to explore the possibility of predicting distance maps using a machine learning approach. For that purpose we build an artificial neural network based on the 2D-RNN adaptive architecture, previously described in [17,39] and further outlined here in Methods and Additional file. The 2D-RNN-based model is used for mapping 2D matrices of variable size into matrices of the same size. The output of the model *O* represents the distance map itself, whereas the input *I* encodes a set of pairwise properties of the residues in the protein (Additional file 1: Figure S1). In particular, the input vector *I*_*j,k*_ associated with *j*^th^ and *k*^th^ residue pair contains: evolutionary information, secondary structure, solvent accessibility and contact density information (Figure 1, Stage 2). The output vector *O*_*j,k*_ represents the predicted distance between the *j*^th^ and *k*^th^ residue pair. To predict *O*_*j,k*_, the 2D-RNN model learns pairwise properties of different parts of the input space i.e. the distance O_*j,k*_ will not depend only on information contained in *I*_*j,k*_ but also to some degree on the *I*_*m≠j,n≠k*_ vectors associated with the properties of all other residue pairs in the protein (Additional file 1: Figure S1).

We construct here two classes of models, a template-based and an *ab initio* model. To dissect the importance of evolutionary information on preserving inter-residue distances in homologous proteins, we also compare *ab initio* models that utilize various types of amino acid information. In particular, we compare performances of the ‘classical’ model which encodes the common 20 types of amino acids, the ‘complementarity’ model restricted only to seven classes of amino acids playing a crucial role in the stability of a protein fold (Methods), and the ‘correlation’ model where amino acid information is augmented by the correlated mutation signal extracted from multiple sequence alignments (MSAs). The correlation model provides the most informative statistics among the three *ab initio* models, and therefore it is expected to outperform the other two. The template-based model is expected to perform substantially better than the *ab initio* models when reliable templates are available, i.e. templates with more than 25-30% sequence identity to the query. All models are trained using a dataset containing 3,645 proteins shorter than 200 residues, described in detail in the Methods section. The models are then tested using a 5-fold cross validation and results obtained are listed in the following.

**Figure 2:**
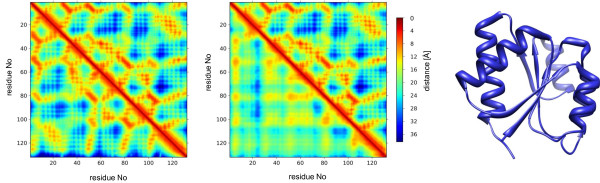
**An example of the distance map prediction.** An example of the template-based (left) and *ab initio* (right) distance map predicted for the protein with PDB ID: 3KHT (145 residues). The best template sequence identity to the query is 24.6%. Residue numbers are given on the axes, whereas the inter-residue distances [Å] are depicted by the colour scheme provided. Average RMSDs of the predicted template-based and *ab initio* maps are 2.86Å and 5.47Å respectively.

In Table 2 we report RMSDs obtained for the *ab initio* and template-based distance predictions as a function of sequence identity to the best template. According to Table 2, the average RMSD between the native and predicted distance maps obtained as outputs of the *ab initio* and template-based classical models are 5.85Å and 3.70Å, respectively. The use of templates improves predictions for every level of sequence identity to the best template, except for the [0, 20%) identity range in which the performances of the two systems are similar. The gain is particularly substantial for higher sequence similarity (40-95%) and exceeds the value of 3Å. An example of the *ab initio* and template-based predicted distance map for a protein with the best template sequence identity of 23.5% is given in Figure 2. The top right of either map depicts a native map, whereas the bottom left represents a predicted map. While the *ab initio* predicted map contains some error areas giving an RMSD between the native and predicted distances of 5.5Å, the template-based distance map correctly reproduces the native map giving an RMSD of 2.9Å.

**Table 2:** Performance of the distance map algorithm

**Seq. id [%]**	**[0, 10)**	**[10,20)**	**[20,30)**	**[30,40)**	**[40,50)**	**[50,60)**	**[60,70)**	**[70,80)**	**[80,90)**	**[90,95)**	**All**
**Model**											
*TB*	5.7±3.4	6.5±3.5	4.4±3.2	3.1±2.1	2.6±1.6	2.4±1.5	2.4±1.3	2.3±1.3	2.5±1.4	2.6±1.9	3.70±2.9
	(7.1)	(7.1)	(4.6)	(3.3)	(3.1)	(2.5)	(2.5)	(2.3)	(2.4)	(2.8)	(4.52)
*AI* classical	5.5±2.5	6.3±2.8	5.9±2.5	5.8±2.6	5.6±1.9	5.7±2.0	5.5±1.9	5.6±2.0	6.0±2.9	6.1±3.2	5.85±2.6
	(7.0)	(6.9)	(6.6)	(6.5)	(6.6)	(6.3)	(6.3)	(6.5)	(6.7)	(6.8)	(6.75)
*AI*	5.5±2.6	6.3±2.8	5.9±2.6	5.8±2.5	5.6±1.9	5.7±2.1	5.5±1.9	5.7±2.0	6.0±3.0	6.1±3.1	5.85±2.6
Compl.	(7.1)	(6.9)	(6.7)	(6.5)	(6.6)	(6.3)	(6.2)	(6.4)	(6.7)	(6.7)	(6.75)
*AI*	5.6±2.6	6.3±2.5	5.9±2.4	5.8±2.4	5.6±1.7	5.6±1.6	5.7±2.1	5.6±1.9	6.2±3.0	6.3±3.4	5.90±2.6
Correl.	(7.1)	(7.0)	(6.7)	(6.3)	(6.6)	(6.3)	(6.6)	(6.6)	(6.8)	(6.9)	(6.81)

RMSD [Å] of *ab initio* (*AI*) and template-based (*TB*) predictions of inter-residue distances as a function of sequence identity to the best template. RMSD is calculated for all residue pairs belonging to the particular protein and then averaged for all proteins in the data set. Values in the brackets are obtained by averaging the obtained RMSDs across the all residue pairs in the dataset.

If one focuses on the value of RMSD between native and predicted distances averaged for all residue pairs in the test dataset (given in brackets in Table 2) and the value of RMSD averaged on a protein level (given without brackets in Table 2), it is obvious that distance-based RMSDs are slightly higher than the corresponding protein-based RMSDs for all levels of sequence identity. This is a consequence of the fact that the prediction capability of the algorithm deteriorates when the length of the protein sequence increases.

We also report in Table 2 the performances of the three *ab initio* models with different contents of leveraged evolutionary information: the classical, the complementarity and the correlation models. According to Table 2, the performances of all three models are undistinguishable and produce an average RMSD of: 5.85Å, 5.85Å and 5.90Å, respectively. Furthermore, the models are also tied for every level of sequence similarity implying that the evolutionary information in terms of classes of amino acids with different physicochemical properties provides sufficient information in predicting inter-residue distances using this machine learning approach.

### Inter-residue separation

**Figure 3:**
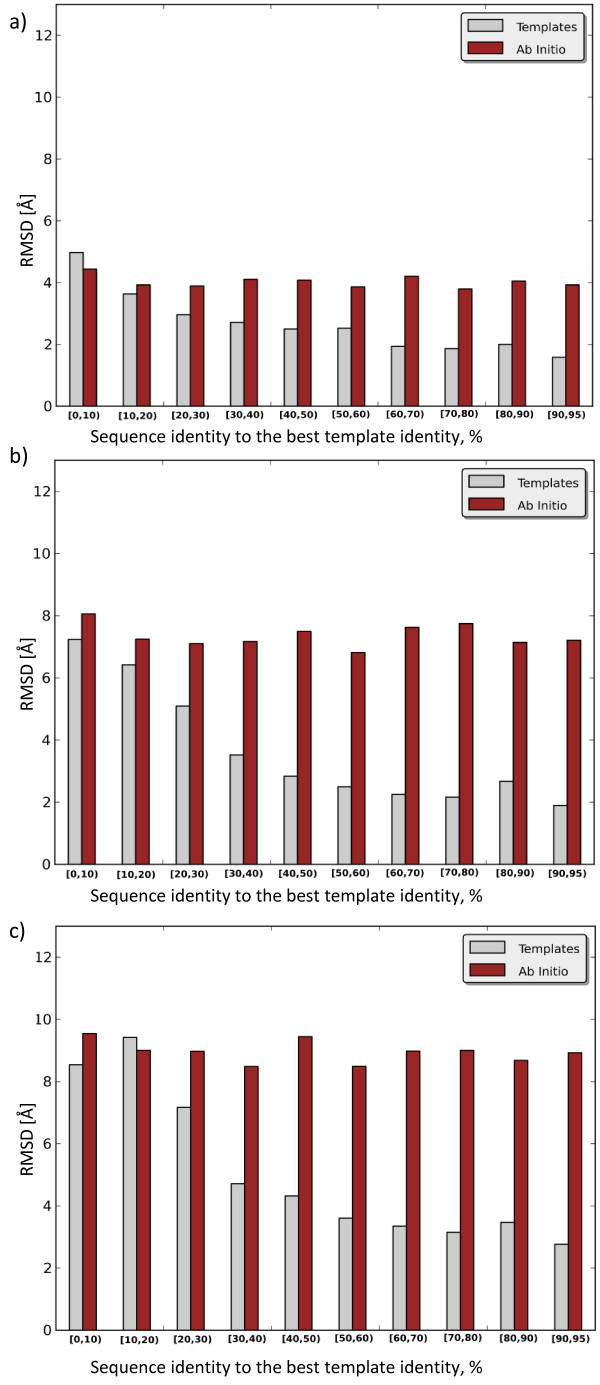
**Distance maps prediction vs. sequence separation.** RMSD [Å] of the classical model predictions for residue pairs with sequence separation **(a)** between 6 and 11 residues **(b)** between 12 and 23 residues **(c)** of more than 23 residues. X-axis represents the sequence identity between the query and the best template.

Beside the overall prediction capability, it is also important to evaluate our model’s ability to predict distances at a specific inter-residue sequence separation. Distances between residues belonging to the same secondary structure element (an α-helix or a β-strand) are much easier to predict than other inter-residue distances in the protein. Accordingly, a β-strand can be recognized in distance profiles by peaks at very short sequence separation (up to 5 residues), whereas an α-helix can be observed in the profiles up to a sequence separation of 20 residues [40]. Therefore, in Figures 3a-c we depict RMSDs of the obtained distances predicted for residue pairs with sequence separations between 6 and 11 residues, between 12 and 23 residues, and 24 residues or more.

According to Figures 3a-c it is evident that distances between neighbouring residues in sequence are predicted more accurately than distances between residues far away in the sequence. In particular, the *ab initio* model produces an overall RMSD of 3.9Å for residues with sequence separation between 6 and 11, an overall RMSD of 5.9Å for residues with sequence separation between 12 and 23, and an overall RMSD of 7.3Å for residues separated by more than 24 amino acids in the sequence. The template-based model improves overall RMSD down to 2.6Å for residues with sequence separation between 6 and 11, to 4.3Å for residues with sequence separation between 12 and 23, and to 6.0Å for residues separated by more than 24 amino acids in the sequence. Moreover, if we compare the performances of the models depending on the sequence identity to the best template, then it is evident that the template-based model outperforms its *ab initio* counterpart for almost all sequence identity ranges and sequence separations. The results between the models are only comparable when the sequence identity to a query is [0, 10%) with the residues being 6 to 11 positions apart, and when the sequence identity to a query is [10, 20%) with the residues more than 24 positions apart in the sequence.

### The template-based model

To quantify the improvement gained when templates are included in this machine learning model, we report the prediction of distances between residue pairs depending on their coverage by the providing templates. Firstly, in Table 3 we report results for only those residues not covered by any template in the dataset. According to Table 3, an average improvement of 1.8Å in RMSD of distance predictions using template-based predictors over the *ab initio* predictors is reported. When the model is able to identify good quality templates, the gain becomes even more obvious with values as large as 4Å when the best template with sequence identity above 90% is identified.

**Table 3:** Performance for non-template regions

**Seq. id [%]**	**[0, 10)**	**[10, 20)**	**[20, 30)**	**[30, 40)**	**[40, 50)**	**[50, 60)**	**[60, 70)**	**[70, 80)**	**[80, 90)**	**[90, 95)**	**All**
**Model**											
TB classical	7.3	8.3	6.8	4.7	6.0	4.0	4.7	4.1	4.8	4.8	5.67
AI classical	7.1	7.5	7.9	7.7	8.8	8.4	7.1	8.5	9.5	9.2	7.46

RMSD [Å] of *ab initio* (*AI*) and template-based (*TB*) predictions of inter-residue distances for non-template regions of the distance map.

In addition, in Table 4 we report the comparison between predictions with the template-based model and a baseline model. The baseline model is built from the same templates used for training purposes. In the first approach, the baseline model simply calculates distances between residues in the best template i.e. the template with the lowest PSI-BLAST e-value. In the second approach, the baseline model is built using all templates and their corresponding weights. The weights depend on templates’ quality and sequence identity to the target. This weighted baseline model corresponds to the way the templates are presented to the neural networks and it’s explained in the Methods section (see Equations 1, 2, 3, 4 and 5). The baseline models are not built using comparative modelling software because this would introduce a different degree of uncertainty depending on the target.

**Table 4:** Performance for template-covered regions

**Seq. id [%]**	**[0, 10)**	**[10, 20)**	**[20, 30)**	**[30, 40)**	**[40, 50)**	**[50, 60)**	**[60, 70)**	**[70, 80)**	**[80, 90)**	**[90, 95)**	**All**
**Model**											
TB classic.	6.7	6.0	3.9	3.0	2.5	1.9	1.8	1.9	1.9	1.9	3.7
Baseline	8.8 (7.3)	9.7 (7.2)	5.2 (4.3)	3.2 (2.9)	2.7 (2.4)	1.9 (1.8)	1.6 (1.7)	1.9 (1.8)	1.8 (1.8)	1.8 (1.8)	5.1 (4.1)

RMSD [Å] of template-based (*TB*) predictions of inter-residue distances for template-covered regions of the distance map. Baseline is a predictor that copies the distances from the best hit template or the weighted templates (given in brackets).

According to Table 4, the overall RMSD obtained using the template-based prediction of distances is 3.7Å, and represents a 1.4Å (0.4Å) improvement over to the best template (the weighted baseline) model. If the available best template is of a high quality (more than 50% of sequence identity), then the predictions between the models become comparable, with the baseline model performing slightly better by increasing the sequence identity. On the other hand, in both the so-called twilight [20, 30%) and midnight zone [0, 20%) of sequence identity, where it is particularly hard to extract information from the templates, the template-based model outperforms the best template hit with a clear margin of up to 3.7Å, and the weighted average model with a clear margin of up to 1.2Å.

### Modelling protein structures using distance maps and CASP9 targets

In the final part of this study we examine the possible application of the template-based and the *ab initio* models of distance maps in the reconstruction of 3D protein structures. To this end, we incorporate the procedure for distance map prediction into a structure prediction pipeline given in Figure 1. The prediction pipeline is modular and includes three steps. In the first step evolutionary information leveraged from the MSA is used to predict several structural features and to generate PDB templates. Among the structural features secondary structure classes (α-helix, β-strand, coil), relative solvent accessibility (surface exposed, buried residue) and reside contacts are predicted. Predictors of the structural features are based on the class of neural networks called bidirectional recurrent neural networks (BRNNs), explained elsewhere [13,41-45]. In the second step, the template-based and the *ab initio* predictor developed in this study are implemented. The predicted distance map output from this step represents a topological representation of the protein 3D structure. Finally, in the last step the actual 3D coordinates of the protein atoms are reconstructed using the restraints provided by the distance map and the basic geometrical rules [15].

As a test dataset in the reconstruction process we use 27 free-modelling and 112 comparative-modelling CASP9 targets [46]. To assess the ability of the presented machine learning approach in reconstructing 3D structures, we benchmark the obtained result on the similar machine learning approach participated in the CASP9 experiment, named Distill. The Distill server predicts 4-class distance maps and employs the reconstruction protocol similar to the protocol explained here. However, the reconstruction protocol implemented in Distill has an additional fragment-based step (see Methods). To benchmark the performance of the distance map approach to the corresponding contact map approach we implement the identical reconstruction algorithm here. The performances of the two algorithms are listed in detail in Additional file 1: Table S3 and summarized here in Table 5.

**Table 5:** Reconstruction of CASP 9 targets

**Maps**	**GDT_TS**	**TM-score**
4-Class (template)	0.61 (0.11, 0.97)	0.66 (0.18, 0.98)
4-Class (*ab initio*)	0.22 (0.09, 0.43)	0.23 (0.12, 0.31)
Distance (template)	0.54 (0.11, 0.91)	0.62 (0.15, 0.91)
Distance (*ab initio*)	0.22 (0.07, 0.44)	0.24 (0.12, 0.43)

The reconstruction of CASP 9 targets using predicted 4-class contact maps and distance maps. Average GDT_TS [fraction] and TM-score, along with their range (min, max) are reported.

According to Additional file 1: Table S3 and Table 5 the reconstruction algorithm that uses distance maps predicted by the template-base predictor reproduces the CASP 9 targets with an average GDT_TS of 53.8% and a TM-score of 0.62. The corresponding 4-class-based predictor produces the structures with an average GDT_TS of 60.9% and a TM-score of 0.66. The results obtained show that the distance map-based reconstruction produces the structures whose quality slightly degrades compared to the corresponding structures obtained by the 4-class map approach. The reported GDT_TS score decreases by 7.1% on average, whereas the TM-score decrease by 0.04 on average. The main reason for the slight decrease in the performance of the distance-based algorithm results from its inability to accurately reproduce structures longer than 200 residues. When targets with sequence length below 200 amino acids are considered, the final results of the two methods become comparable with an average TM-score of 0.56 and 0.55 for the 4-class based predictor and distance-map predictor, respectively (Additional file 1: Table S5).

When the reconstruction algorithm uses distance maps predicted by the *ab initio* predictor, the performance of the model significantly drops as expected (Additional file 1: Table S3 and Table 5). Both the distance-based and the 4-class-based reconstruction protocols give similarly low performances: GDT_TS = 22%, TM-score = 0.24 when distance maps are used; and GDT_TS = 22%, TM-score = 0.23 when 4-class maps are used. These results show that the current distance/contact map machine learning approach is not able to reliably reproduce protein structures using only protein sequence information coupled with basic geometrical rules, and should be complemented in the future by more complex reconstruction protocol.

**Figure 4:**
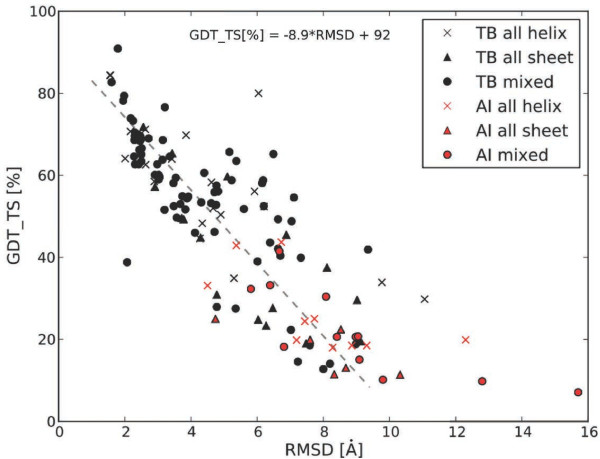
**Correlation between the quality of the predicted distance maps and the quality of the reconstructed structures exemplified on the CASP9 targets.** The x-axis depicts the RMSD [Å] between the predicted and native distance maps of the CASP9 targets. The y-axis depicts the GDT_TS [%] score between the reconstructed and native CASP9 targets. The correlation coefficient between the RMSD and GDT_TS values is 0.78. *Ab initio* maps are given in red, whereas template-based maps are given in black. Proteins with different secondary structure content are shown separately.

Finally, we try to establish a correlation between the quality of reconstruction with the quality of a predicted distance map. This is similar to the approach summarized in Table 1. However, instead of generating native distance maps with certain amount of noise, we use the distance maps predicted for the CASP9 targets and their corresponding reconstructed structures. In Figure 4, we show the dependence of the RMSD between the predicted and native distance maps, and the GDT_TS score of the reconstructed structures. According to Figure 4, there is a strong correlation between the quality of the distance maps and the quality of the reconstructions with a Pearson correlation coefficient of 0.78. This correlation was independent of the secondary structure content. For values of RMSD bellow 9Å this dependence is linear, whereas for values of RMSD above 9Å the reconstruction protocol produces structures of poor quality. Similar to the previous conclusion (see Table 1), it is evident that only the distance maps predicted with the precision better than RMSD = 6Å can produce meaningful structures (GDT_TS > 0.4) using this simple reconstruction protocol. Distance maps of this quality used with a more advanced reconstruction protocol can represent a valuable approach in future protein structure prediction efforts.

## Discussion

We have explored the possibility of predicting protein inter-residue distances using amino-acid information on its own, or complemented by structural templates. The prediction of inter-residue distances and other continuous structural features in proteins in general has been avoided in the past due to complexity of the problem in comparison to the traditional methods that predict their discrete counterparts. Only recently, have new models that successfully predict continuous backbone torsion angles emerged [47,48]. These models have shown that the accurate prediction of continuous backbone torsion angles can be obtained and hence more accurate sampling of the protein conformational space can be achieved. In light of this, we have developed a novel machine learning pipeline for high-throughput prediction of protein distance maps, based on the similar machine learning approach previously developed for contact map predictions [30].

We show that when a physically realizable distance map is used as target, even a simple geometry-based reconstruction algorithm is able to reproduce a 3D structure that is almost identical to the target. In contrast, a full set of discrete restraints, in terms of binary and 4-class distance maps, used with the same reconstruction protocol yield a 3D structure with considerably lower resolution. When non-physical target maps are used, the quality of the reconstructed structure gets degraded when the amount of noise in the map increases. However, the distance map has been shown to be more resistant to noise than initially envisioned. Even when a random error of ±6Å is introduced into the distance map, the reconstructed protein structure is more accurate than the corresponding structures reconstructed from the binary and 4-class map, giving an average RMSD to the target below 2Å.

We have presented two systems for the prediction of distance maps: the *ab initio* and the template-based system trained on protein sequences with less than 200 residues. The *ab initio* system was implemented using various degrees of evolutionary content: 7 classes of amino acids with different physicochemical properties, 20 common amino acid, and 20 common amino acids complemented with correlated mutations in the MSA. The performances of all three *ab initio* models are comparable implying that the evolutionary information in terms of 7 classes of amino acids with different physicochemical properties provides sufficient information in predicting inter-residue distances using this machine learning approach. Furthermore, the template-based system was capable of exploiting both sequence and structure information even in cases of dubious homology. In both twilight [20, 30%) and midnight zone [0, 20%) of sequence identity, where it is particularly hard to extract information from the templates, the template-based model outperforms the best template hit with a clear margin of up to 3.7Å, and outperforms the weighted average model with a clear margin of up to 1.2Å

Finally, we have shown that predicted real-value distances do not lack the ability to reconstruct near-native structures compared to the predicted contacts. When distance maps were tested on the CASP9 targets sequences, the performance of the distance-based algorithm was comparable to the performance of the 4-class-based approach, implemented in the Distill server, for targets shorter than 200 residues. However, in the current implementation this approach is not suitable for the targets longer than 200 residues. This is expected as distance maps encode more structural information than their discrete counterparts, and their complexity precludes their application on long sequences.

The methodology presented here in its current implementation is not as accurate as the existing techniques that utilize complex force field functions, statistical constraints or additional NMR spectra. However, it is important to note here that the presented method does not aim to replace the existing state of the art models. Instead, our goal was to explore the possibility of predicting protein continuous features, as inter-residue distances, using an efficient machine learning approach. Information on inter-residue and inter-atomic distances in proteins represents valuable information in structural biology, best exemplified by the shown direct dependence of NMR chemical shifts on inter-atomic distances [49]. Therefore, possible application of this method is aimed not only in structure prediction protocols, but also as an additional tool to complement experimental data.

## Conclusions

In this work we explore the possibility of predicting protein inter-residue distances and further using them as constraints in the protein reconstruction procedure. The distance map representation of protein topology can tolerate a large amount of noise and still lead to correct 3D structures even when a simple reconstruction protocol is employed. Therefore, the methodology we presented, if complemented by more complex reconstruction protocols, may represent a possible path to improve machine-learning algorithms for 3D protein structure prediction.

## Methods

### Distance map prediction using 2D-Recursive Neural Network

The artificial neural networks we used for predicting distance maps are based on the general-purpose 2D-RNN adaptive architecture previously described in [17,39] and further outlined here in SI. A description of the architecture of the 2D-RNN together with details on the learning algorithm we employ is also provided in SI. 2D-RNN-based models are used for mapping 2D matrices of variable size into matrices of the same size. Here, the output of the model *O* represents the distance map itself, whereas the input *I* encodes a set of pairwise properties of the residues in the protein (Additional file 1: Figure S1).

The input vector *I*_*j,k*_ associated with the *j*^th^ and *k*^th^ residue pair contains evolutionary information, secondary structure, solvent accessibility and contact density information. The frequencies of amino acids observed in the two columns, *j* and *k,* of the MSA are used as an evolutionary input to the network, therefore, representing two 20-dimensional probability vectors. Structural information in the form of standard secondary structure classes (α-helix, β-sheet, random coil) is encoded using two 3-dimensional vectors, whereas relative solvent accessibility (2 classes: buried, 0-25%; and exposed, 25-100%) and contact density (4 classes) are encoded using two 2-dimensional and two 4-dimensional vectors, respectively. In total, a vector of 58 units is used as an input to the *ab initio* model of the distance map prediction.

The model that encodes 20 common types of amino acids is termed the ‘classical’ model here. In addition to this classical model, two additional models with different evolutionary contents are created. The first, so-called “complementarity” model restricts the input to seven classes of amino acids that are expected to be relevant to the stability of the fold. The complementarity model clusters 20 amino acids into 7 classes based on their structural and physicochemical properties: (i) hydrophobic (A, F, I, L, M, V), (ii) polar (N, Q, S, T, W, Y), (iii) negatively charged (D, E), (iv) positively charged (H, K, R), (v) cysteine (C), (vi) glycine (G) and (vii) proline (P). In the second model, so called “correlation” model, amino acid information is augmented by the correlated mutation signal (1 unit) extracted from the MSA. Correlated mutations are calculated using the PAM70 substitution matrix and Göbel’s algorithm [50], in which completely conserved positions and the positions with > 20% gaps are discarded from the analysis.

In the template-based model an additional 2-dimensional vector extracted from template PDB profiles is appended to the input vector, similarly to [30]. The first unit in this vector encodes the weighted average distance from the templates:

(1) $$d_{\mathrm{jk}}^{T}=\frac{\sum_{p=1}^{M}w_{p}d_{\mathrm{jk}}^{p}}{\sum_{p=1}^{M}w_{p}}$$

where *w*_*p*_ represents the weight attributed to the *p* template. The weight *w*_*p*_ depends on the template’s quality, *q*_*p*_, and its sequence identity, *id*_*p*_, to the target sequence:

(2) $$w_{p}=q_{p}id_{p}^{3}$$

The template quality further depends on the nature of the 3D structure (X-ray, NMR), its resolution and R-factor [51], which for X-ray structures is given by:

(3) $$q_{p}\left[X_{\text{ray}}\right]=\frac{1}{\text{resolution}\left[Å\right]+\frac{R_{\text{factor}}}{20}}$$

and for NMR structures:

(4) $$q_{p}\left[\text{NMR}\right]=\frac{1}{\text{resolution}\left[Å\right]+\frac{R_{\text{factor}}}{10}}$$

Taking into account the cube of the sequence identity between the query and the template in Equation 3 allows us to favour those distances extracted from good templates over the distances calculated from low-similarity templates.

Finally, the last unit in the input vector encodes the weighted average of the template coverage and it is given by:

(5) $$c_{j,k}^{T}=\frac{\sum_{p=1}^{M}w_{p}c_{p}}{\sum_{p=1}^{M}w_{p}}$$

where *c*_*p*_ represents the coverage of the query by the template, i.e. the fractions of the non-gaps in the alignment. The template-based vector defined this way performed better than the number of alternatives in the preliminary testing (data not shown) and is used in the template-based models of distance maps.

The input vector $I_{j,k}^{\text{filter}}$ provided to the filtering NN contains the predicted distance *d*_*jk*_ obtained from the previous 2D-RNN network (1 unit), sequence separation between residues *j* and *k* (1 unit), the protein sequence length (1 unit) and global information extracted from the predicted distance map (15 units). The global information contains the average distance between all pairs of amino acid (*m*, *n*) within the segments *j* − 5 ≤ *m* ≤ *j* + 5 and *k* − 5 ≤ *n* ≤ *k* + 5 In addition to the average distance of this 11×11 residue patch positioned around the (*j*, *k*) residue pair, the average distances of 14 additional patches are also provided to the network by keeping the same separation between the pairs of residues, as in [52].

### Learning and initialization

The 2D-RNNs composing the distance map predictors are trained by minimizing the squared error between the output and the target distances. To avoid large plateaux in the error function at the beginning of the training, a modified form of the gradient-descent algorithm is used. This algorithm employs a piecewise linear function in three different ranges for the network update weights, and is discussed in detail in [39]. The transfer functions in all network units are implemented using the *tanh* function. We adopt a hybrid between on-line and batch training with 1,450 batch blocks per training set, i.e. two proteins per a batch. That is, the weights of all networks are updated based on the gradient computed on groups of two proteins. To prevent the error to decrease monotonically, the training set is shuffled at the beginning of each epoch. If the error does not decrease for 50 consecutive epochs, the learning rate is divided by 2. Prior to learning, the weights in each unit in all neural networks are randomly initialized. Their standard deviations are controlled in a flexible way, so as to avoid any bias and ensure that the expected total input into each unit remains approximately in the same range.

Due to the large number of training instances and limited computational power/time, all systems are trained in 5-fold cross validation. Each of the five networks is trained for 1000 epochs by saving the parameters every 5 epochs. For each network the last three saved models are combined in the single predictor. Finally, all 5 networks are combined in a single system. This is known to slightly improve the performance over individual models [39].

### Reconstruction algorithm

The reconstruction algorithm of protein *C*_*α*_ -traces is organized into two sequential phases, as described in detail in [53]. Shortly, in the first phase a random structure is generated by adding *C*_*α*_ positions until the whole backbone is produced. The bonds of adjacent *C*_*α*_ atoms in this phase are added in a random direction with the lengths restricted to lie in the interval 3.803±0.07 Å using uniform distribution. The positions of the *C*_*α*_ atoms belonging to a helix structure are modelled using the coordinates of the ideal helix with random orientation. In the last phase, the algorithm refines the initial structure by optimizing the pseudo-energy function using local moves and simulating annealing [15]. The moves we adopt displace a single residue at a time, and keep its distances to its neighbours constant.

The pseudo-energy function used here is shaped to encode the constraints represented by the distance map and various geometrical limitations. Let *S*_*n*_ = {*r*_*i*_}_*i* = 1 … *n*_ be a sequence of *n* 3D coordinates, with *r*_*i*_ = (*x*_*i*_, *y*_*i*_, *z*_*i*_) being the coordinates of the *i*^*th*^*C*_∝_ atom of the current protein conformation and *d*_*ij*_ = |*r*_*i*_ − *r*_*j*_| the distance between the atoms *i* and *j*. Then, the set of constraints guiding the reconstruction of the protein structure can be written by ℳ=D∪ℬ∪C∪S. The first set of constraints  comes from the predicted distance map $D_{S_{n}}={\left\{d_{\mathrm{ij}}^{\text{map}}\right\}}_{i<j},$ containing *n* × (*n* − 1) mutual distances between *C*_∝_ atoms. The distances $d_{\mathrm{ij}}^{\mathrm{map}}$ are obtained as outputs from the second step of the overall pipeline (Figure 1). The rest of the geometricconstraints include: $B=\left\{d_{\mathrm{ij}}\;\in \left[D_{B}\;-0.07,\;D_{B}+0.07\right],\left|i\;-\;j\right|=1,\;D_{B}=3.803Å\right\}$ which limits neighbouring *C*_*α*_ distances; $C=\left\{d_{\mathrm{ij}}>D_{\mathrm{CL}},\left|i-j\right|>1,D_{\mathrm{CL}}=4Å\right\}$ which defines clashes between residues; and $S=\left\{D_{\min}^{\text{stand}}<d^{\text{strand}}<D_{\text{max}}^{\text{stand}},D_{\text{min}}^{\text{stand}}=l\times \left(3.436–0.05107\times l\right)-0.04\times l^{2},D_{\min}^{\mathrm{stand}}=l\times \left(3.436–0.05107\times l\right)+0.04\times l^{2}\right\}$ which defines the dependence of the distance between the first and the last residue in the β-strand *d*^*strand*^ on the amino acid length of *l* of the strand. Using these constraints the pseudo-energy function can be written by:

(6) $$\begin{array}{l} E\left(S_{n},ℳ\right)=\alpha_{0}\left\{\alpha_{1}\sum_{i<j}\left|d_{\mathrm{ij}}-d_{\mathrm{ij}}^{\text{map}}\right|+\sum_{\left|i-j\right|=1}{\left(d_{\mathrm{ij}}-D_{B}\right)}^{2}\right\}\\ \;\;\;+\alpha_{2}\sum_{\left|i-j\right|>1}10^{\left|d_{\mathrm{ij}}-D_{\mathrm{CL}}\right|}\\ \;\;\;+\alpha_{3}\sum_{\text{strands}}{\left(d^{\text{strand}}-D^{\text{strand}}\right)}^{2} \end{array}$$

In all the experiments, we run the annealing protocol for 10,000 × protein length iterations, in each of which the perturbation of a single residue is attempted. Pseudo-energy parameters are set to, *α*_0_ = 0.2 *α*_1_ = 0.025 (distance penalty), *α*_2_ = 0.5 (clashes) and *α*_3_ = 2.0 (strand length), so that the conformational search is biased towards the generation of compact, clash-free structures with the recommended length of β-strands and with *C*_∝_ distances approaching to distances provided by the distance map.

A distance map contains no information about chirality. When an overall structure is reconstructed, the mirror-image structure is equally legitimate, having the same distance map. Therefore, in the final step we generate the mirror image of the reconstructed structure and refine it for additional 5,000 iterations. The choice of the final reconstructed structure depends on the pseudo-energy penalty needed for the original and mirror image reconstructed 3D structure (Equation 6).

In addition to this simple geometry-based reconstruction algorithm, we use a fragment-based reconstruction to predict the structures of CASP9 targets from non-native 4-class maps and distance maps. In the fragment-based reconstruction [54] implemented here, for each protein segment of length 9, 50 candidate structures in the PDB are identified using the fold recognition algorithm described in [55]. A move consists in swapping a segment at a random position with another (random) one in the list. Since segment lengths are generally not the same, mutual distances between any two residues in the protein are affected by a move. Moves are accepted or rejected based on the same pseudo-energy function as in the previous protocol (Equation 6) and the simulating annealing protocol for 20,000 iterations. Lastly, the mirror image of the reconstructed structure is generated, a brief further reconstruction is attempted and its fitness is assessed. Given that segments from the PDB incorporate chirality information, we observe that, in the majority of cases, the correct mirror image is selected directly based on fitness.

### Datasets

The dataset used to train and test the predictors is extracted from the October 2009 25% pdb_select list containing 4,818 proteins [56]. Since the training is computationally demanding (and its complexity quadratic in the protein length) we created a reduced version of the dataset by excluding proteins longer than 200 residues. The final dataset contains 3,645 proteins with 360,971 residues and 21,918,875 residue pairs (Additional file 1: Table S2). All systems are trained in 5-fold cross validation by splitting the dataset into 5 approximately equal folds. Inter-residue distances used for training are measured between C_α_ atoms, and their distribution is plotted in Additional file 1: Figure S2. If we include only the distances between residues separated by at least 2 amino acids in the sequence, then, it becomes clear from Additional file 1: Figure S2 that the majority of data (76%) are distributed in the range [10Å, 30Å] with a mean value of 20.7Å.

Secondary structure and relative solvent accessibility of each residue are assigned using DSSP [57], whereas contact density is calculated as in [44]. True structural information is used for training of both the *ab initio* and the template-based models. For testing purposes, we use predictions from in-house servers [42,44,45] to predict secondary structure, solvent accessibility and contact density, respectively.

Evolutionary information in the form of amino acid probability vectors, amino acid classes and correlated mutations are calculated from MSAs. The alignments for the proteins in the training/test dataset are extracted from the non-redundant (NR) database. The alignments are generated by three runs of position specific iterative BLAST (PSI-BLAST) [58] with parameters *b=3,000*, *e =10*^*-3*^ and *h=10*^*-10*^.

To generate the structural templates for a protein, we run PSI-BLAST against the PDB (available on April 30th 2008) using the position specific scoring matrix (PSSM) generated during the alignment process. We deliberately use a high expectation parameter (*e=10*) to include hits that are beyond the usual comparative modelling scope (*e<0.01*). Finally, in order to avoid perfect templates coming from PDB resubmissions of the same structure and close homologues, we exclude those templates whose sequence similarity exceeds 95% over the whole query.

The distribution of the sequence identity to the average/best template identity is given in Additional file 1: Figure S3. The average identity for all templates, not surprisingly, is generally low with a median of 20% identity. Although the distribution is not uniform, all identity intervals are adequately represented: 37% of all proteins have the best hit with less than 20% sequence identity (midnight zone), the best hit of 21% proteins is between 20-30% sequence identity (twilight zone), and for the rest of 42%, close homologues can be found with sequence identity in the interval 30-95%.

## Competing interests

The authors declare that they have no competing interests.

## Authors’ contributions

PK designed, trained and tested the predictor of distance maps. CM designed the fragment-based reconstruction protocol. GT and PV provided training datasets. IW designed the predictor of contact maps. GP designed the RNN architecture and was included in the reconstruction of CASP9 targets. The manuscript was written by PK and GP, and read and approved by other authors.

## Supplementary Material

Additional file 1 Supplementary information. Click here for file
