# Supplementary material for: Toward an accurate prediction of inter-residue distances in proteins using 2D recursive neural networks
Source: BMC Bioinformatics. 2014 Jan 10;15:6. doi: 10.1186/1471-2105-15-6 (PMC3893389; doi:10.1186/1471-2105-15-6)
Supplement: Additional file 1 — Supplementary information. [file 1471-2105-15-6-S1.docx]

# Additional file

## Distance map prediction using 2D-Recursive Neural Network

2D-RNN-based models are used for mapping 2D matrices of variable size into matrices of the same size. Here, the output of the model *O* represents the distance map itself whereas the input *I* encodes a set of pairwise properties of the residues in the protein (Figure S1).

Let the indices *j* and *k* represent residue positions in the protein sequence, then the input-output mapping between the input vector *I_j,k_* and its corresponding distance *O_j,k_* is given in the form:

$$O_{j,k}=\mathcal{N}^{O}\left( I_{j,k},H_{j,k}^{SE}, H_{j,k}^{SW}, H_{j,k}^{NW}, H_{j,k}^{NE} \right)$$

$$H_{j,k}^{SE}=\mathcal{N}^{SE}(I_{j,k},H_{j-1,k}^{SE}, H_{j,k+1}^{SE})$$

$H_{j,k}^{SW}=\mathcal{N}^{SW}(I_{j,k},H_{j+1,k}^{SW}, H_{j,k+1}^{SW})$ (1)

$$H_{j,k}^{NW}=\mathcal{N}^{NW}(I_{j,k},H_{j+1,k}^{NW}, H_{j,k-1}^{NW})$$

$$H_{j,k}^{NE}=\mathcal{N}^{NE}\left( I_{j,k},H_{j-1,k}^{NE}, H_{j,k-1}^{NE} \right)$$

$$j, k=1\ldots N$$

Hidden unit vectors *H_j,k_* in *Eq.1* represent contextual memories that encode information about different parts of the input map. Likewise the hidden context which keeps track of all amino acids before and after the provided input amino acid in predicting structural features from the protein sequence [1-6], hidden units specialize in memorizing the pairwise properties of different parts of the input map: SE, SW, NW, NE, as depicted in Figure S1 (right).

Each of the five functions (the output update 𝒩^O^ and the four lateral update functions 𝒩^SE^, 𝒩^SW^, 𝒩^NW^, 𝒩^NE^) are parameterized using an independent two layered feed-forward network [7]. In order to reduce the number of free parameters, the stationarity is assumed for all residue pairs i.e. the five neural networks share the same parameters across all residue pairs (j=1...N, k=1..N).

The distance map obtained as an output of this 2D-RNN is often inherently local, i.e. lacks ability to reproduce short distances between distant residues in the sequence. Therefore, in order to predict more “physical” distance map, i.e. a map that can be embedded into a 3D structure, the distance map is further filtered using another 2D-RNN, called filtering NN, as explained in [8]. The filtering NN is input more global information i.e. not only the distance between the particular residues *j* and *k*, but also the distances between residues further away in the sequence. That way we are able to enhance the quality of the final map, mostly by more accurate predictions of the distances away from the main diagonal. Short distances between residues further away in the sequence are both harder to predict and more informative in determining the overall topology of the protein structure.

## Architectures

We use two 2D-RNNs, the prediction and the filtering network, each one consisting of five feed-forward neural networks as in *Eq.1*. The hidden contextual networks ($\mathcal{N}^{i}, i=SE, SW, NE, NW$) contain a hidden layer with *N_hh_* hidden units and an output layer with *N_ho_* units. With an input size of $\left| I \right|$, the total number of inputs to the hidden layer of the hidden contextual network is $\left| I \right|+2N_{ho}$. Thus, including the bias term in each layer, the total number of parameters in the hidden contextual network is $\left( \left| I \right|+2N_{ho} \right)\times N_{hh}+N_{hh}+N_{hh}\times N_{ho}+N_{ho}$. The output network $\mathcal{N}^{O}$ contains $\left| I \right|$ regular inputs and $N_{ho}$ contextual outputs from each of the four hidden contextual networks, resulting in the input size of $\left| I \right|+4N_{ho}$ units. Furthermore, the output network has a single hidden layer with *N_h_* hidden units which gives the total number of $\left( \left| I \right|+4N_{ho} \right)\times N_{h}+N_{h}+N_{h}+1$ parameters in the output network. The number of parameters used in all the three models is summarized in Table S1.

## Additional file 1: Figure S1

General layout of the 2D-RNN used for predicting distance maps. The nodes are arranged on a square lattice in one input plane, one output plane and four hidden planes. The hidden planes contain horizontal directed edges associated with the square lattice and oriented in the direction of one of the four possible cardinal corners: SE, SW, NW, NE. Additional vertical directed edges connect the plains in columns, with the input plane being connected to all hidden planes and the output plane, and the hidden planes being connected to the output plane. The input variable *I_i,j_* represents the vector of inputs at position (*i, j*), whereas the variable *O_i,j_* denotes the corresponding output. The four hidden vectors represent contextual memories of the different parts of the input plane and their graphical representation is given on the right.

Adapted with permission from [7].


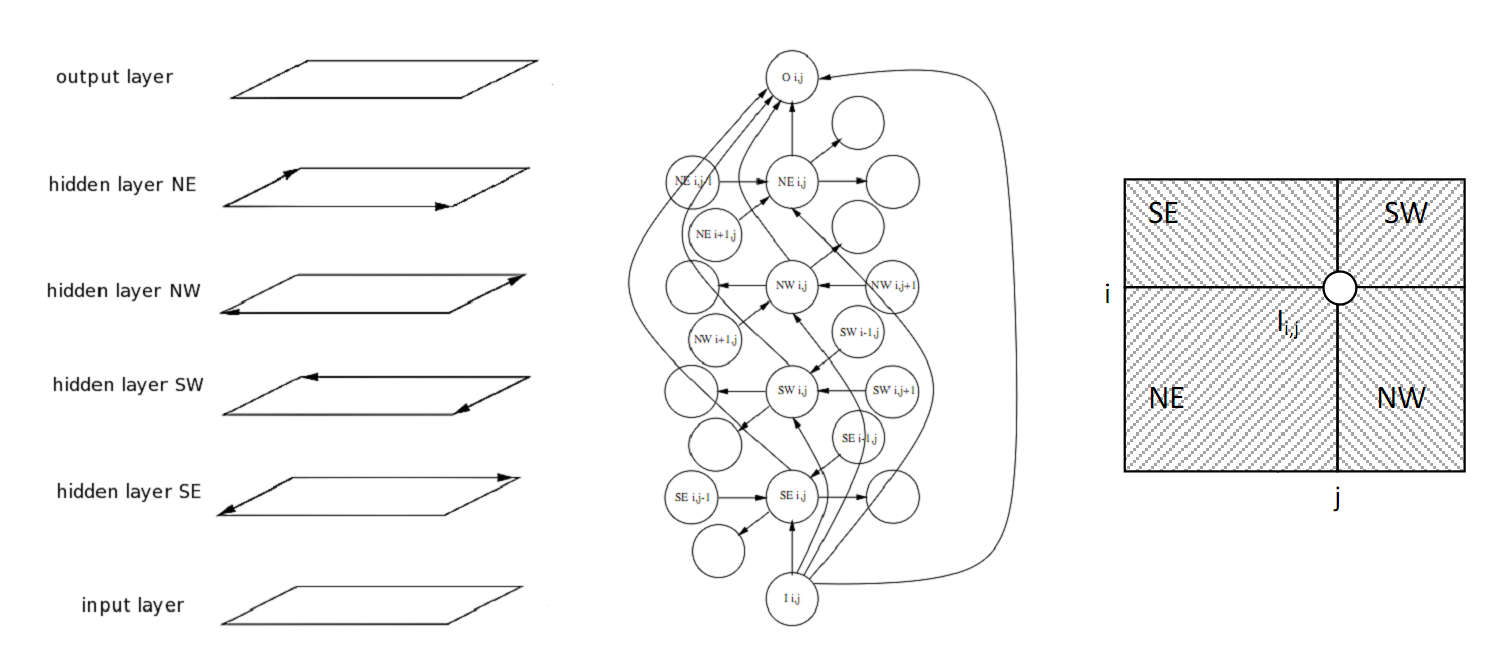


## Additional file 1: Figure S2

The distribution of distances used for the training and test purpose. Only the distances between residues separated by at least 2 amino acids are included in the figure. The peaks at short-distance ranges (3-11 Å) are mainly due to local structural elements, such as α-helices and β-sheets. The vertical line indicates an average distance of the distribution, being 20.7 Å.


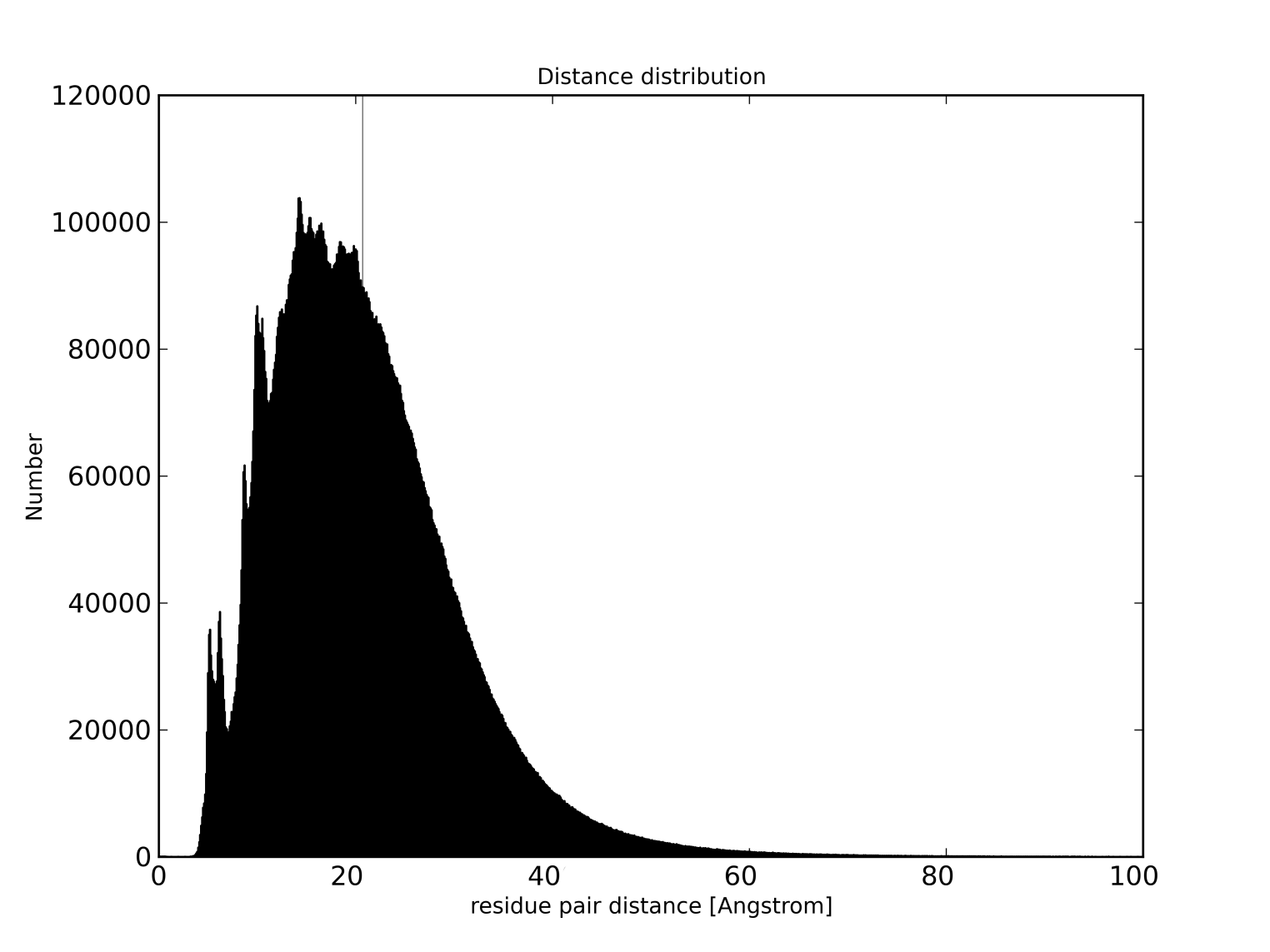


## Additional file 1: Figure S3

The distribution of sequence identity to the average/best template identity in the dataset. Hits above 95% of sequence similarity are excluded.

**
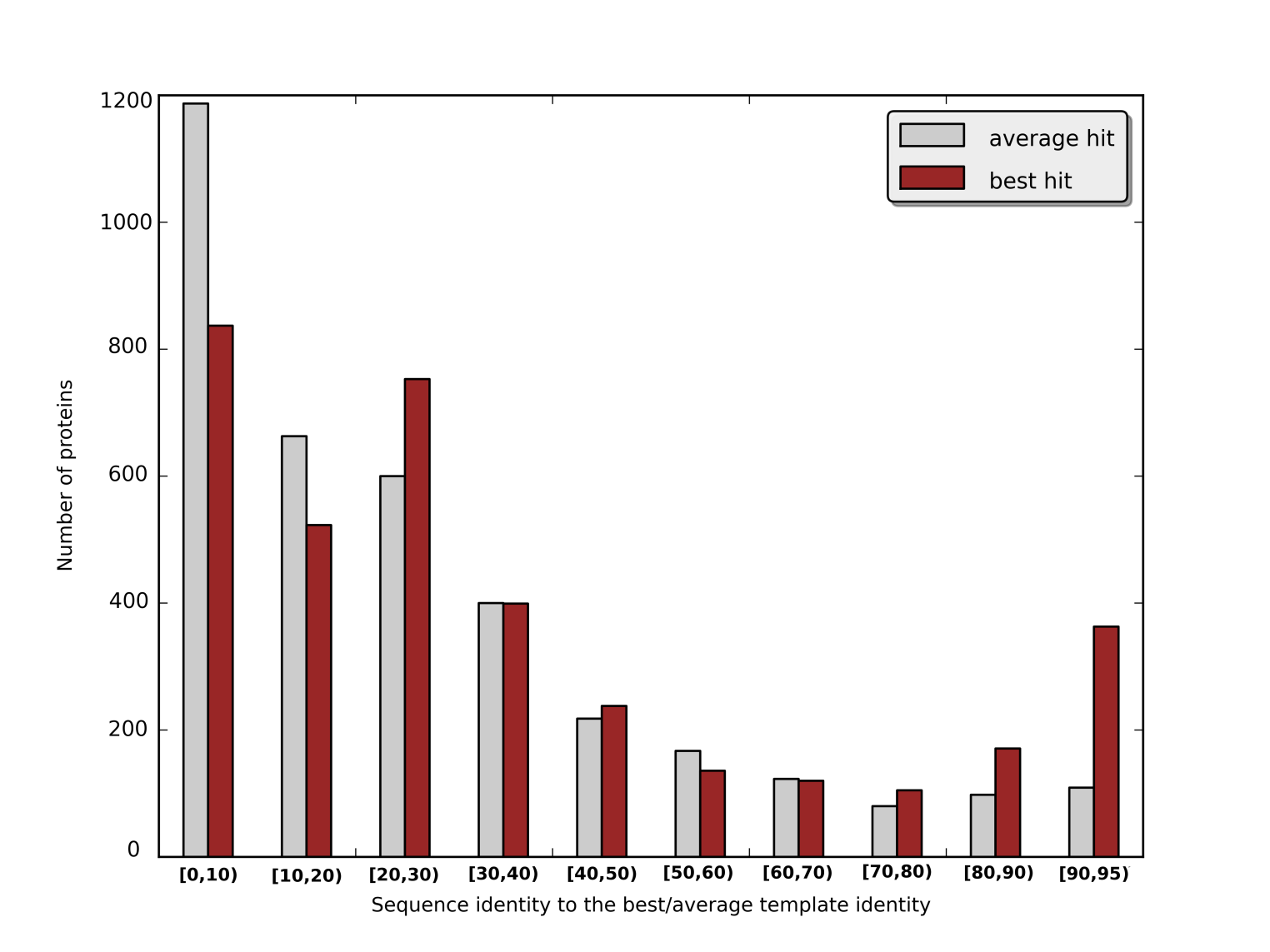
**

## Additional file 1: Table S1

Model architectures used in distance map predictions. *N_h_* is the number of units in the hidden output network, whereas *N_hh_* and *N_ho_* represent the number of hidden and output units in the hidden contextual networks.

| model | | **prediction network** | | | | | **filtering network** | | | | | total |
| --- | --- | --- | --- | --- | --- | --- | --- | --- | --- | --- | --- | --- |
|  |  | I | Nhh | Nho | Nh | parameters | I | Nhh | Nho | Nh | parameters |  |
| **ab initio** | **classical** | 58 | 14 | 14 | 14 | 1428 | 18 | 7 | 7 | 7 | 533 | 1961 |
|  | **complementarity** | 32 | 14 | 14 | 14 | 1064 | 18 | 7 | 7 | 7 | 533 | 1597 |
|  | **correlation** | 59 | 14 | 14 | 14 | 1442 | 18 | 7 | 7 | 7 | 533 | 1975 |
| **template-based**  **classical** | | 60 | 14 | 14 | 14 | 1456 | 18 | 7 | 7 | 7 | 533 | 1989 |

## Additional file 1: Table S2

The number of proteins and residue pairs (given in brackets) used in the particular training/test fold.

| fold  dataset | **fold 0** | **fold 1** | **fold 2** | **fold 3** | **fold 4** |
| --- | --- | --- | --- | --- | --- |
| **training** | 2911  (17,390,365) | 2939  (17,722,182) | 2919  (17,502,806) | 2899  (17,387,127) | 2912  (17,673,020) |
| **test** | 734  (4,528,510) | 706  (4,196,693) | 726  (4,416,069) | 746  (4,531,748) | 733  (4,245,855) |

## Additional file 1: Table S3

Reconstruction of CASP9 targets using predicted 4-class contact maps and distance maps. Targets reconstructed with the *ab initio* models are colored red.

| **target/method** | 4-class map GDT_TS [%] | 4-class map TM-score [%] | distance GDT_TS [%] | distance TM-score [%] |
| --- | --- | --- | --- | --- |
| **T0515-D1** | 64.8 | 85.2 | 49.7 | 77.5 |
| **T0516-D1** | 72.9 | 85.3 | 64.0 | 82.0 |
| **T0517-D1** | 58.1 | 66.2 | 54.6 | 65.5 |
| **T0518-D1** | 68.5 | 77.7 | 58.1 | 74.2 |
| **T0520-D1** | 76.4 | 83.5 | 68.8 | 79.5 |
| **T0521-D1** | 68.6 | 68.7 | 52.0 | 54.9 |
| **T0521-D2** | 71.7 | 66.4 | 70.7 | 67.5 |
| **T0522-D1** | 93.3 | 95.2 | 78.2 | 86.2 |
| **T0523-D1** | 75.6 | 78.7 | 65.1 | 70.7 |
| **T0524-D1** | 63.4 | 84.0 | 54.8 | 80.7 |
| **T0525-D1** | 64.6 | 79.3 | 56.1 | 75.0 |
| **T0526-D1** | 68.3 | 86.5 | 57.2 | 81.4 |
| **T0527-D1** | 78.0 | 76.9 | 66.2 | 68.9 |
| **T0528-D1** | 77.8 | 88.7 | 64.6 | 82.6 |
| **T0528-D2** | 58.1 | 68.6 | 51.6 | 65.7 |
| **T0529-D1** | 8.9 | 17.6 | 7.1 | 15.8 |
| **T0529-D2** | 11.9 | 18.2 | 12.8 | 21.9 |
| **T0530-D1** | 68.7 | 67.1 | 63.8 | 63.5 |
| **T0531-D1** | 31.4 | 23.4 | 33.2 | 27.7 |
| **T0532-D1** | 54.8 | 79.8 | 46.0 | 75.6 |
| **T0533-D1** | 68.7 | 81.3 | 53.0 | 71.2 |
| **T0533-D2** | 80.7 | 81.6 | 68.4 | 70.0 |
| **T0534-D1** | 15.4 | 19.1 | 18.5 | 23.3 |
| **T0534-D2** | 17.7 | 22.9 | 18.5 | 23.8 |
| **T0536-D1** | 73.1 | 75.6 | 68.6 | 71.7 |
| **T0537-D1** | 11.7 | 23.9 | 19.9 | 37.4 |
| **T0537-D2** | 37.1 | 14.3 | 32.3 | 13.6 |
| **T0538-D1** | 80.6 | 71.1 | 71.2 | 60.4 |
| **T0539-D1** | 71.6 | 67.6 | 58.1 | 52.3 |
| **T0540-D1** | 30.8 | 29.6 | 23.3 | 22.1 |
| **T0541-D1** | 73.0 | 76.9 | 65.4 | 71.7 |
| **T0542-D1** | 59.7 | 77.6 | 49.3 | 71.0 |
| **T0542-D2** | 78.5 | 89.8 | 51.7 | 76.3 |
| **T0543-D1** | 61.8 | 37.8 | 38.8 | 23.0 |
| **T0543-D2** | 52.7 | 38.7 | 73.9 | 59.9 |
| **T0543-D3** | 46.3 | 57.8 | 41.9 | 69.2 |
| **T0543-D4** | 46.2 | 63.5 | 39.9 | 62.6 |
| **T0544-D1** | 25.5 | 31.3 | 18.2 | 21.3 |
| **T0545-D1** | 67.1 | 73.4 | 60.1 | 69.3 |
| **T0547-D1** | 18.9 | 23.0 | 14.1 | 19.0 |
| **T0547-D2** | 68.6 | 86.9 | 59.0 | 81.2 |
| **T0547-D3** | 16.7 | 17.0 | 18.0 | 15.7 |
| **T0547-D4** | 43.3 | 30.2 | 42.9 | 30.3 |
| **T0548-D1** | 42.1 | 23.6 | 42.1 | 31.7 |
| **T0548-D2** | 42.9 | 33.2 | 44.6 | 38.0 |
| **T0550-D1** | 16.0 | 21.8 | 13.1 | 17.0 |
| **T0550-D2** | 13.5 | 19.2 | 11.4 | 17.7 |
| **T0551-D1** | 38.8 | 26.3 | 34.9 | 29.3 |
| **T0552-D1** | 53.7 | 45.8 | 49.3 | 45.1 |
| **T0553-D1** | 43.2 | 31.4 | 43.7 | 35.2 |
| **T0553-D2** | 29.9 | 25.2 | 33.1 | 31.1 |
| **T0555-D1** | 18.1 | 19.9 | 19.8 | 22.2 |
| **T0557-D1** | 46.2 | 51.0 | 52.5 | 57.9 |
| **T0558-D1** | 47.4 | 71.3 | 44.8 | 67.6 |
| **T0559-D1** | 75.3 | 70.6 | 62.7 | 57.6 |
| **T0560-D1** | 79.6 | 71.3 | 69.5 | 62.0 |
| **T0562-D1** | 19.9 | 22.5 | 18.9 | 21.6 |
| **T0563-D1** | 60.0 | 74.0 | 54.9 | 72.8 |
| **T0564-D1** | 63.9 | 52.3 | 52.5 | 43.0 |
| **T0565-D1** | 48.7 | 68.6 | 43.6 | 64.3 |
| **T0566-D1** | 69.4 | 78.4 | 53.2 | 65.7 |
| **T0567-D1** | 76.4 | 82.0 | 69.6 | 77.4 |
| **T0568-D1** | 19.3 | 21.9 | 19.0 | 22.7 |
| **T0569-D1** | 25.9 | 21.4 | 27.6 | 24.0 |
| **T0570-D1** | 75.6 | 88.4 | 62.7 | 82.6 |
| **T0571-D1** | 16.3 | 24.0 | 11.5 | 16.6 |
| **T0571-D2** | 20.1 | 25.3 | 22.4 | 26.9 |
| **T0572-D1** | 21.8 | 21.8 | 19.5 | 18.0 |
| **T0573-D1** | 68.5 | 84.5 | 59.6 | 80.6 |
| **T0574-D1** | 34.8 | 36.3 | 24.8 | 26.7 |
| **T0575-D1** | 73.8 | 67.9 | 69.8 | 66.4 |
| **T0575-D2** | 70.6 | 77.7 | 62.6 | 69.3 |
| **T0576-D1** | 18.8 | 20.6 | 18.6 | 22.8 |
| **T0578-D1** | 19.7 | 24.2 | 20.6 | 24.3 |
| **T0579-D1** | 26.6 | 20.7 | 29.6 | 24.7 |
| **T0579-D2** | 35.5 | 24.0 | 30.9 | 26.0 |
| **T0580-D1** | 81.4 | 85.0 | 69.0 | 74.6 |
| **T0581-D1** | 23.8 | 24.5 | 20.7 | 21.0 |
| **T0582-D1** | 54.9 | 61.3 | 59.7 | 67.2 |
| **T0582-D2** | 47.7 | 49.1 | 45.5 | 46.6 |
| **T0584-D1** | 64.9 | 84.4 | 50.4 | 71.4 |
| **T0585-D1** | 68.7 | 79.5 | 60.6 | 75.0 |
| **T0586-D1** | 90.3 | 87.3 | 86.6 | 87.3 |
| **T0586-D2** | 91 | 77.9 | 74.1 | 68.3 |
| **T0588-D1** | 35.8 | 52.2 | 33.9 | 55.8 |
| **T0590-D1** | 77 | 74.2 | 80.1 | 74.9 |
| **T0591-D1** | 74.0 | 90.4 | 64.6 | 87.5 |
| **T0592-D1** | 72.2 | 75.4 | 63.5 | 69.9 |
| **T0593-D1** | 61.5 | 77.3 | 60.1 | 78.7 |
| **T0594-D1** | 79.8 | 87.0 | 65.7 | 77.2 |
| **T0596-D1** | 90.0 | 84.0 | 84.4 | 74.9 |
| **T0596-D2** | 60.3 | 68.2 | 58.5 | 66.7 |
| **T0597-D1** | 57.1 | 77.9 | 53.4 | 76.7 |
| **T0598-D1** | 45.4 | 50.2 | 40.4 | 47.5 |
| **T0599-D1** | 73.3 | 90.7 | 54.4 | 79.2 |
| **T0600-D1** | 71.1 | 63.3 | 73.3 | 67.4 |
| **T0600-D2** | 78.1 | 58.3 | 71.8 | 56.9 |
| **T0601-D1** | 71.3 | 92.0 | 55.9 | 85.8 |
| **T0602-D1** | 88.1 | 81.3 | 80.0 | 73.8 |
| **T0603-D1** | 47.9 | 69.1 | 51.8 | 72.6 |
| **T0604-D1** | 23.1 | 19.6 | 20.6 | 19.9 |
| **T0604-D2** | 36.7 | 47.2 | 22.3 | 32.4 |
| **T0604-D3** | 15.1 | 23.3 | 10.2 | 17.9 |
| **T0605-D1** | 51.5 | 44.2 | 56.1 | 41.1 |
| **T0606-D1** | 41.4 | 45.5 | 39.0 | 43.5 |
| **T0607-D1** | 57.4 | 83.0 | 46.2 | 77.5 |
| **T0608-D1** | 23.3 | 21.1 | 19.9 | 17.0 |
| **T0608-D2** | 46.1 | 54.7 | 48.8 | 57.4 |
| **T0609-D1** | 56.6 | 79.7 | 49.6 | 76.4 |
| **T0610-D1** | 65.7 | 78.3 | 59.4 | 73.7 |
| **T0611-D1** | 90.5 | 84.0 | 84.4 | 77.1 |
| **T0611-D2** | 43.2 | 53.1 | 48.3 | 58.5 |
| **T0612-D1** | 36.3 | 37.2 | 37.5 | 42.2 |
| **T0613-D1** | 84.0 | 94.2 | 66.6 | 87.0 |
| **T0615-D1** | 62.7 | 67.7 | 58.3 | 67.5 |
| **T0616-D1** | 25.7 | 24.7 | 30.4 | 30.0 |
| **T0617-D1** | 76.8 | 81.9 | 69.9 | 72.6 |
| **T0618-D1** | 22.4 | 26.8 | 25.0 | 31.3 |
| **T0620-D1** | 70.1 | 83.1 | 57.5 | 78.6 |
| **T0621-D1** | 12.4 | 16.9 | 15.1 | 23.0 |
| **T0622-D1** | 56.1 | 61.1 | 58.8 | 63.2 |
| **T0623-D1** | 61.5 | 72.7 | 52.8 | 67.2 |
| **T0624-D1** | 22.8 | 17.6 | 25.0 | 20.7 |
| **T0625-D1** | 65.8 | 78.3 | 58.8 | 74.2 |
| **T0626-D1** | 85.0 | 94.5 | 63.4 | 85.6 |
| **T0627-D1** | 57.6 | 75.4 | 52.4 | 72.8 |
| **T0628-D1** | 18.9 | 24.4 | 14.6 | 17.7 |
| **T0628-D2** | 39.0 | 48.6 | 27.9 | 34.5 |
| **T0629-D1** | 59.2 | 47.4 | 62.7 | 55.0 |
| **T0629-D2** | 9.9 | 12.1 | 9.8 | 12.4 |
| **T0630-D1** | 36.3 | 37.9 | 27.5 | 30.1 |
| **T0632-D1** | 89.2 | 90.9 | 82.7 | 86.9 |
| **T0634-D1** | 89.2 | 89.9 | 79.4 | 83.0 |
| **T0635-D1** | 97.0 | 97.8 | 90.9 | 90.7 |
| **T0637-D1** | 24.6 | 24.6 | 24.4 | 27.9 |
| **T0638-D1** | 75.0 | 87.0 | 65.2 | 80.5 |
| **T0639-D1** | 28.6 | 29.5 | 41.5 | 42.8 |
| **T0640-D1** | 77.9 | 88.6 | 70.4 | 85.9 |
| **T0641-D1** | 76.6 | 91.2 | 68.6 | 87.6 |
| **T0643-D1** | 31.5 | 25.5 | 29.8 | 25.1 |
| **TBM** | **60.9** | **66.1** | **53.8** | **61.5** |
| ***Ab initio*** | **22.0** | **22.6** | **22.4** | **23.7** |
| **ALL** | **53.15** | **56.81** | **47.52** | **53.91** |

## Additional file 1: Table S4

Reconstruction of the CASP9 targets with sequence length below 200 residues using predicted 4-class contact maps and distance maps.

| **target/method** | 4-class map GDT_TS [%] | 4-class map TM-score [%] | distance GDT_TS [%] | distance TM-score [%] |
| --- | --- | --- | --- | --- |
| **T0517-D1** | 58.1 | 66.2 | 54.6 | 65.5 |
| **T0520-D1** | 76.4 | 83.5 | 68.8 | 79.5 |
| **T0521-D2** | 71.7 | 66.4 | 70.7 | 67.5 |
| **T0522-D1** | 93.3 | 95.2 | 78.2 | 86.2 |
| **T0523-D1** | 75.6 | 78.7 | 65.1 | 70.7 |
| **T0527-D1** | 78 | 76.9 | 66.2 | 68.9 |
| **T0529-D2** | 11.9 | 18.2 | 12.8 | 21.9 |
| **T0530-D1** | 68.7 | 67.1 | 63.8 | 63.5 |
| **T0536-D1** | 73.1 | 75.6 | 68.6 | 71.7 |
| **T0538-D1** | 80.6 | 71.1 | 71.2 | 60.4 |
| **T0540-D1** | 30.8 | 29.6 | 23.3 | 22.1 |
| **T0541-D1** | 73 | 76.9 | 65.4 | 71.7 |
| **T0543-D2** | 52.7 | 38.7 | 73.9 | 59.9 |
| **T0543-D3** | 46.3 | 57.8 | 41.9 | 69.2 |
| **T0545-D1** | 67.1 | 73.4 | 60.1 | 69.3 |
| **T0548-D1** | 42.1 | 23.6 | 42.1 | 31.7 |
| **T0548-D2** | 42.9 | 33.2 | 44.6 | 38 |
| **T0551-D1** | 38.8 | 26.3 | 34.9 | 29.3 |
| **T0552-D1** | 53.7 | 45.8 | 49.3 | 45.1 |
| **T0557-D1** | 46.2 | 51 | 52.5 | 57.9 |
| **T0560-D1** | 79.6 | 71.3 | 69.5 | 62 |
| **T0562-D1** | 19.9 | 22.5 | 18.9 | 21.6 |
| **T0564-D1** | 63.9 | 52.3 | 52.5 | 43 |
| **T0566-D1** | 69.4 | 78.4 | 53.2 | 65.7 |
| **T0567-D1** | 76.4 | 82 | 69.6 | 77.4 |
| **T0568-D1** | 19.3 | 21.9 | 19 | 22.7 |
| **T0569-D1** | 25.9 | 21.4 | 27.6 | 24 |
| **T0572-D1** | 21.8 | 21.8 | 19.5 | 18 |
| **T0574-D1** | 34.8 | 36.3 | 24.8 | 26.7 |
| **T0576-D1** | 18.8 | 20.6 | 18.6 | 22.8 |
| **T0579-D1** | 26.6 | 20.7 | 29.6 | 24.7 |
| **T0579-D2** | 35.5 | 24 | 30.9 | 26 |
| **T0580-D1** | 81.4 | 85 | 69 | 74.6 |
| **T0582-D1** | 54.9 | 61.3 | 59.7 | 67.2 |
| **T0582-D2** | 47.7 | 49.1 | 45.5 | 46.6 |
| **T0586-D1** | 90.3 | 87.3 | 86.6 | 87.3 |
| **T0590-D1** | 77 | 74.2 | 80.1 | 74.9 |
| **T0592-D1** | 72.2 | 75.4 | 63.5 | 69.9 |
| **T0593-D1** | 61.5 | 77.3 | 60.1 | 78.7 |
| **T0594-D1** | 79.8 | 87 | 65.7 | 77.2 |
| **T0596-D1** | 90 | 84 | 84.4 | 74.9 |
| **T0596-D2** | 60.3 | 68.2 | 58.5 | 66.7 |
| **T0598-D1** | 45.4 | 50.2 | 40.4 | 47.5 |
| **T0600-D1** | 71.1 | 63.3 | 73.3 | 67.4 |
| **T0600-D2** | 78.1 | 58.3 | 71.8 | 56.9 |
| **T0602-D1** | 88.1 | 81.3 | 80 | 73.8 |
| **T0605-D1** | 51.5 | 44.2 | 56.1 | 41.1 |
| **T0606-D1** | 41.4 | 45.5 | 39 | 43.5 |
| **T0608-D2** | 46.1 | 54.7 | 48.8 | 57.4 |
| **T0610-D1** | 65.7 | 78.3 | 59.4 | 73.7 |
| **T0612-D1** | 36.3 | 37.2 | 37.5 | 42.2 |
| **T0615-D1** | 62.7 | 67.7 | 58.3 | 67.5 |
| **T0617-D1** | 76.8 | 81.9 | 69.9 | 72.6 |
| **T0622-D1** | 56.1 | 61.1 | 58.8 | 63.2 |
| **T0623-D1** | 61.5 | 72.7 | 52.8 | 67.2 |
| **T0629-D1** | 59.2 | 47.4 | 62.7 | 55 |
| **T0630-D1** | 36.3 | 37.9 | 27.5 | 30.1 |
| **T0632-D1** | 89.2 | 90.9 | 82.7 | 86.9 |
| **T0634-D1** | 89.2 | 89.9 | 79.4 | 83 |
| **T0635-D1** | 97 | 97.8 | 90.9 | 90.7 |
| **T0639-D1** | 28.6 | 29.5 | 41.5 | 42.8 |
| **T0643-D1** | 31.5 | 25.5 | 29.8 | 25.1 |
| **TBM** | **57.9** | **57.8** | **54.4** | **56.3** |

# References

1. Baldi P, Brunak S, Frasconi P, Soda G, Pollastri G: **Exploiting the past and the future in protein secondary structure prediction** *Bioinformatics* 1999, **15**(11):937-946.

2. Pollastri G, McLysaght A: **Porter: a new, accurate server for protein secondary structure prediction**. *Bioinformatics* 2005, **21**(8):1719-1720.

3. Pollastri G, Przybylski D, Rost B, Baldi P: **Improving the Prediction of Protein Secondary Structure in Three and Eight Classes Using Recurrent Neural Networks and Profiles**. *PROTEINS: Structure, Function, and Genetics* 2002, **47**:228-235.

4. Vullo A, Walsh I, Pollastri G: **A two-stage approach for improved prediction of residue contact maps**. *BMC Bioinformatics* 2006, **7**.

5. Pollastri G, Baldi P, Fariselli P, Casadio R: **Prediction of Coordination Number and Relative Solvent Accessibility in Proteins**. *Proteins: Structure, Function, and Bioinformatics* 2002, **47**(2):142-153.

6. Pollastri G, Martin A, Mooney C, Vullo A: **Accurate prediction of protein secondary structure and solvent accessibility by consensus combiners of sequence and structure information**. *BMC Bioinformatics* 2007, **8**:201.

7. Baldi P, Pollastri G: **The Principled Design of Large-Scale Recursive Neural Network Architectures-DAG-RNNs and the Protein Structure Prediction Problem**. *Journal of Machine Learning Research* 2003, **4**:575-602.

8. Martin A, Bau D, Vullo A, Walsh I, Pollastri G: **Long-range information and physicality constraints improve predicted protein contact maps**. *Journal of Bioinformatics and Computational Biology* 2008, **6**(5):1001-1020.
